# Supplementary figures and images for: Optimization and action mechanism of pollutant removal performance of unsaturated vertical flow constructed wetland (UVFCW) driven by substained-release carbon source
Source: PeerJ. 2025 Jan 16;13:e18819. doi: 10.7717/peerj.18819 (PMC11742248; doi:10.7717/peerj.18819)

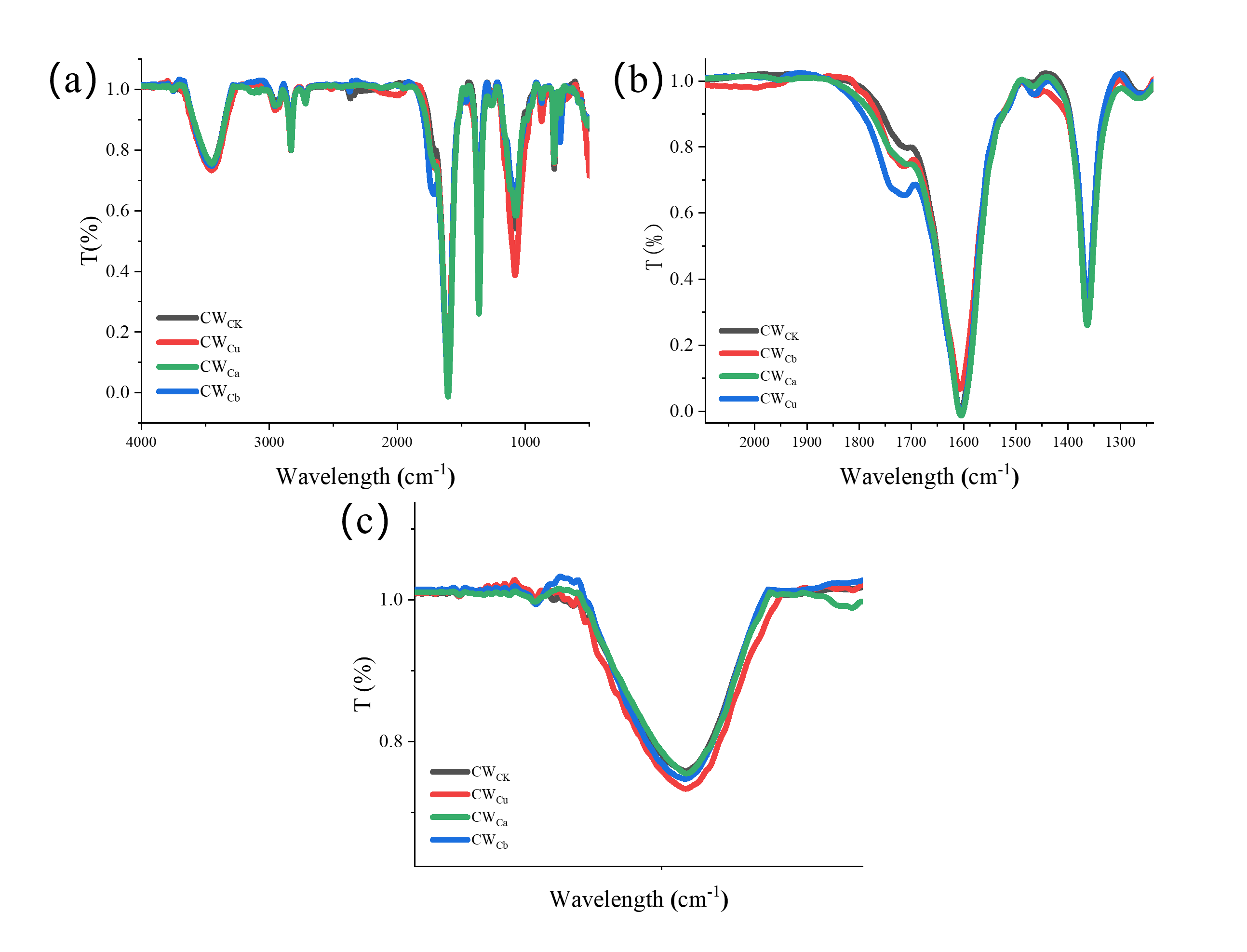

Supplement: Supplemental Information 4 — CWCu represents uniform slow-release carbon source, CWCa represents upper slow-release carbon source, CWCb represents lower slow-release carbon source, CWCK represents original slow-release carbon source. [file peerj-13-18819-s004.png]
